# Supplementary material for: Are carbon emissions trading and green financial instruments synergistic? -Comprehensive quantitative research based on content analysis
Source: PLoS One. 2024 Mar 7;19(3):e0298601. doi: 10.1371/journal.pone.0298601 (PMC10919601; doi:10.1371/journal.pone.0298601)
Supplement: S1 Data — (ZIP) [file pone.0298601.s001.zip › Data/地级市Z_F工作报告.zip!/║■▒▒/╛ú╓▌╩╨/└┤╫╘╣1⁄2╓┌║┼í╛┬φ┐╦╩2╛▌═°í┐_2019.docx]

各位代表：

现在，我代表市人民政府向大会报告工作，请予审议，并请各位政协委员和列席人员提出意见。

一、2018年工作回顾

过去一年，是荆州攻坚克难、砥砺前行的一年。在省委、省政府和市委的坚强领导下，在市人大和市政协的监督支持下，我们团结和依靠全市人民，深入贯彻习近平新时代中国特色社会主义思想，积极应对经济下行压力，加快推动新旧动能转换，保持了稳中有进、进中向好的良好发展态势。

这一年，习近平总书记亲临我市、考察荆江，对荆州共抓大保护工作给予肯定，极大鼓舞了全市上下干事创业的斗志和士气，坚定了我们转型赶超、加快复兴的信心和决心。

这一年，省委、省政府先后两次召开现场办公会，研究支持荆州推进长江大保护和高质量发展，明确把江陵煤电港化产业园上升为省级战略，为荆州发展注入了强动力、提供了硬支撑。

这一年，市委审时度势、长远谋划，作出了牢记嘱托、担当作为、奋力谱写新时代荆州高质量发展新篇章的重大决策。一年来，全市上下同心同向、奋发进取，地区生产总值突破2000亿元，地方财政总收入超过200亿元，“互联网+放管服”改革领跑全省，高质量发展浓厚氛围正在加速形成。

这一年，我们经受住重重困难和挑战，取得了发展的新成果、新成效。全年地区生产总值增长7.5%，固定资产投资增长11%，规模以上工业增加值增长6.7%，社会消费品零售总额增长11.1%，地方一般公共预算收入增长9.6%，城乡居民人均可支配收入分别增长8.7%、8.3%。

回顾过去一年，我们主要做了以下工作。

一是致力强产业，提升经济质量。

工业经济增效提质。六大支柱产业加速壮大，电子信息产业产值较上年增长4.6倍。新增规模以上工业企业67家。新增省级隐形冠军企业66家、高新技术企业45家、两化融合试点示范企业26家。白云边税收突破10亿元。五方光电、亿钧耀能荣获工信部“绿色工厂”称号。四机赛瓦油气井封层桥塞入围全国第三批制造业单项冠军产品。久隆汽车动力转向器荣获中国驰名商标。菲利华获评全国年度质量标杆企业。凯乐科技跻身中国上市公司500强。

农业优势巩固提升。新建高标准农田40万亩，新增稻虾共作面积45.8万亩、再生稻面积36.5万亩。新增规模以上农产品加工企业35家，各类农业新型经营主体总量达到4.9万个。新增“三品一标”标志产品63个。荆州高新区晋级国家级高新区，袁隆平院士工作站挂牌。小龙虾产量达到35万吨。监利县蝉联全国小龙虾销量冠军，洪湖市获评“中国小龙虾第一名城”。公安县、洪湖市成功创建省首批农产品质量安全县。公安县获评“江南葡萄第一县”。石首市获评“国家西甜瓜产业技术体系先进示范县”。

现代服务业发展提速。全域旅游加速推进。纪南文旅区完成项目投资110亿元。古城5A创建稳步实施。小梅沙海洋馆开放营业。洈水国家湿地公园获批。旅游综合收入316亿元、增长21%。商贸流通日趋活跃。新增限上商贸企业147家，网商数量突破1万家。荆沙铁路完成货运量408万吨，创历史新高。多层次资本市场建设加快。桐林天然气在香港联交所上市，省级上市后备“银种子”企业达到23家。银行存贷款增量、增幅均保持全省前三位。金融生态环境综合考核连续排名市州第一。

二是致力添动能，培育后劲增量。

重大项目快速推进。100个省、市重点项目建设加快，山鹰纸业、盐卡港综合物流园等项目年度投资计划超额完成，长利玻璃三期、能特维E等项目竣工投产。中心城区政府投资项目完成投资138.5亿元、是上年的2.3倍。实施水利补短板工程，长湖、洪湖围堤加固工程完成投资总量的80%，洪湖东分块蓄洪工程进展顺利。

大量的工程项目上马，刷新了城市颜值，提升了城市竞争力，同时也给市民生活带来了一些不便和干扰，广大群众给予了我们充分的理解和极大的包容。

招商引资成效明显。新引进亿元以上项目485个。吉利新能源动力电池及模组、中材建筑产业园等5个过50亿元项目成功引进。航天科工、中国供销、中农联控股等32家世界和国内500强、央企、上市公司先后进驻。美国天纳克、日本凯迩必、土耳其施坦达、美德合资伟速达、韩国三立等14个外资项目落户荆州。

对外开放步伐加快。国际贸易“单一窗口”企业入驻率和使用率达到85%，企业通关时间较上年压缩三分之一。外贸出口创历史新高，出口总量15.7亿美元、增长29.2%。新增出口企业42家，出口过亿美元企业达到4家。与希腊伊利奥普里斯市、韩国江陵市共建友好城市。

三是致力抓改革，积聚发展能量。

2018年，是改革开放40周年。我们坚持把改革作为破解发展瓶颈、优化发展环境的首要抓手，着力推动重点领域和关键环节改革，为荆州后发赶超汇聚了新的动力。

放管服改革走在全省前列。主动争取“互联网+政务服务”改革纳入省级试点，在全省率先实现省、市、县、乡、村五级“一网联通”。智慧荆州建设加快，“荆州E家”APP开通上线，“一窗受理”集成服务正式运行。行政审批流程进一步优化，“一次办好”事项占市级政务服务事项的90%以上，100个高频事项实现“零见面”审批。“证照分离”改革全面推开，“多证合一”事项由33项增至39项，新企业开办时间由13个工作日压缩至4个工作日。新增市场主体6.7万户、增长7.7%。“放管服”改革成效第三方评估市直全覆盖，工作经验在国务院门户网转载。获评第八届环球总评榜“中国营商环境质量十佳城市”。

供给侧结构性改革不断深化。“三去一降一补”深入推进，去产能、去库存任务全面完成，水、电、气等资源要素成本和融资、涉企税费、制度性交易成本呈下降趋势，累计为企业减负25亿元。产业发展市级统筹体制改革成效显现，“一区多园”工业产值达到全市规模以上工业企业总产值的42.4%。江陵县经济开发区完成扩区调区。农村集体产权制度改革稳步推进，“公安三社”“石首富集体现象”等典型经验在全省交流。

其他领域改革稳步推进。首创长江岸线管理员制度。组建荆州城发集团、荆州开发集团和荆州融资担保集团。新落地PPP项目15个，PPP模式经验在全国交流。税收征管体制改革平稳落地。国有林场改革通过省级验收。医药卫生体制改革扎实推进，分级诊疗制探索起步，县域医共体建设加快。国有农场、城市管理、不动产登记、社会事业和文化体制等领域改革取得新进展。

四是致力打基础，拓展空间容量。

城市建设力度加大。建设复兴大道等六纵八横14条城市主干道98公里。古城内环道改造完成，凤凰路西延伸线、三湾路等建成通车。中心医院新院建设进展顺利，一医新门诊大楼主体工程完工。改造、新建截污管网78公里。持续推进棚改清零，改造棚户区24344套。

大交通格局加快形成。在建高速公路273公里，居全省第一。318国道纪南文旅区段主体工程完工。洪湖赤壁等4座长江大桥稳步推进。荆州民用机场进入施工高峰期。蒙华铁路荆州段开始铺轨。松滋车阳河二期等项目加快推进。普通公路“建养一体化”投资额全省第一。建成“455”安防工程3984公里。新改扩建农村公路2126公里。

乡村振兴迈出坚实步伐。引进“三乡工程”项目416个、总投资85亿元。建设美丽农村路1000公里，荆州区、江陵县获评全省“四好农村路”示范县。农村电网建设投资7.8亿元。完成造林64.1万亩，松滋市、石首市获评省级森林城市。“421工程”加快推进。万全镇获评全国农业产业示范强镇，闸口镇获评全国稻渔生态种养示范镇。三湖农场获评省级特色小镇。八岭山镇等3个乡镇获评省级森林城镇，久合垸等6个乡镇获评省级生态乡镇，监利县望江村等53个行政村获评省级生态村。

五是致力增福祉，彰显民生份量。

生态保护持续加强。长江大保护十大标志性战役全面启动。129处无证非法码头整治到位。87处入河排污口整改提升。10个集中式饮用水源地全部达标。14个地表水国家考核断面达标率提高到85.7%。四湖流域断面监测水质合格率、优良率均提高一倍。“四个三重大生态工程”加快推进。提标改造城市生活污水处理厂10座，新改建乡镇生活污水厂和管网项目104个。建成农户无害化厕所9.4万座，精准灭荒6102亩。扎实推进“两减一控”，农药、化肥使用量分别减少3.9%、2.1%。秸秆露天禁烧工作综合考评排名全省第一，空气质量持续改善，中心城区pm10、pm2.5累计浓度分别下降6.5%、12.5%。

精准脱贫扎实深入。统筹21.6亿元用于精准扶贫。发放小额贷款6.6亿元支持产业扶贫。新建、改扩建贫困村通村公路161.5公里。解决1.8万名贫困人口安全饮水问题。建档立卡贫困户基本医保和大病保险全覆盖，实现先诊疗、后付费和“一站式”报销，贫困人口年度负担不超过5000元。贫困户危房改造和易地搬迁年度任务全面完成。教育资助政策全面落实。保障农村低保和特困供养人员14.7万人，支出社会救助金4.5亿元。53个贫困村、48767人脱贫出列。

社会保障和社会事业全面进步。新增城镇就业7.2万人。社会保险参保人数达到1006万人次。发放公积金个人贷款17.6亿元。新增城市社区居家养老服务中心43个、农村社区老年人互助照料活动中心189个。长江大学入围“双一流”建设高校，参与研究项目荣获国家科学技术进步奖一等奖。成功举办首届荆楚文化旅游节、第二届荆州国际马拉松和第五届市运会，获评全国青少年校园足球改革试验区。汉剧《优孟衣冠》、歌剧《有爱才有家》、红色经典《洪湖赤卫队》在全国各地巡演。楚式漆器髹饰技艺入选第一批国家传统工艺振兴项目。五城同创稳步推进。成功创建“中华诗词之市”“中国楹联文化城市”。血吸虫传播阻断达标考核通过省级验收。出生人口性别比回归正常水平。第四次全国经济普查稳步推进。安全生产事故起数和死亡人数双下降。食品药品安全监管得到加强。拖欠农民工工资问题得到有效治理。“七五”普法中期检查获得好评。人民调解工作卓有成效。“先手行动”扎实开展。化解信访积案560件。“扫黑除恶”专项斗争深入开展，长江生态保卫战强力推进，“3356”社会治安防控体系进一步健全，社会大局保持和谐稳定。

国防动员、国防教育、国家安全、人民防空、民兵预备役工作得到加强。应急管理、防震减灾、对口支援、台湾事务、外事侨务、民族宗教、红十字会、公益慈善等工作取得新成绩，机关事务管理、物价、农垦、移民、烟草、气象、档案、社科、邮政、供销、史志、保密和无线电等工作取得新进步。

各位代表！抓落实是全部政府工作的精髓，提效能是衡量政府工作的重要标尺。一年来，我们坚持党对政府工作的全面领导，不断强化全面从严治党主体责任，严格落实中央八项规定精神，精准实施目标管理、环节管理、过程管理，政府工作效能得到有效提升。自觉运用法治思维和法治方式化解矛盾、解决问题、推动工作。主动接受人大监督、政协民主监督、社会监督和舆论监督。办理人大建议150件、满意率100%，政协提案272件、满意率99.6%。强化监察监督和审计问责，深入开展形式主义、官僚主义问题集中整治年活动，查处问题578个、1350人，政府系统作风建设得到加强。

各位代表！过去一年，政府工作取得的一切成绩，是党中央、国务院和省委、省政府正确领导的结果，是市委团结带领全市人民凝心聚力、奋力拼搏的结果，是市人大、市政协有效监督、大力支持的结果，是社会各界关心帮助、热情参与的结果。在此，我代表市人民政府，向全市广大干部群众，向驻荆部队、武警官兵，向各民主党派、工商联、无党派人士、各人民团体和社会各界人士，致以崇高的敬意！向所有关心、支持、参与荆州改革开放和现代化建设的中外朋友，表示衷心的感谢！

各位代表！在肯定成绩的同时，我们也清醒地认识到，我市经济社会发展还存在一些突出困难和问题。一是发展速度仍然不快，固定资产投资、规模以上工业增加值增速没有达到预期。二是生态环保任务艰巨，尤其是水环境欠账多、治理难度大。三是民生领域还有不少短板，优质医疗、公平教育等供需矛盾突出，中心城区交通拥堵和停车难的问题日益凸显。四是发展环境有待进一步改善，一些部门和工作人员担当不够、作风不实、效能不高，等等。对这些问题，我们一定高度重视、认真面对、精准施策，切实加以解决。

二、2019年重点工作

各位代表！今年是新中国成立70周年，是决胜全面建成小康社会第一个百年奋斗目标的关键之年。

从宏观经济形势看，我国外部发展环境深刻变化，经济下行压力加大，高质量发展面临诸多挑战，我们必须保持定力、稳中求进，以进促稳、以进应变。从湖北区域发展态势看，各地竞相比拼，不进则退、慢进亦退，我们必须向标兵看齐，争先进位、竞以求胜，以实际行动回报省委、省政府和全市人民的殷切期望。从当前荆州的市情看，国家战略叠加汇集，省级支持力度加大，机遇当前、时不我待，我们必须把握机遇、担当作为、乘势而上，不负时代赋予的历史责任和重大使命。

今年政府工作的指导思想是：高举习近平新时代中国特色社会主义思想伟大旗帜，深入学习宣传贯彻党的十九大精神和习近平总书记视察湖北重要讲话精神，认真落实省委十一届四次全会、市委五届七次全会精神，统筹推进“五位一体”总体布局，协调推进“四个全面”战略布局，坚持稳中求进工作总基调，坚持新发展理念，坚持高质量发展，坚持以供给侧结构性改革为主线，坚持深化市场化改革、扩大高水平开放，继续打好“三大攻坚战”，积极融入全省“一芯两带三区”区域和产业发展战略，推动转型赶超、加快荆州复兴，奋力打造“万里长江、美在荆江”新画卷。

今年经济社会发展的主要预期目标是：地区生产总值增长7.5%-8%，固定资产投资增长11.5%，规模以上工业增加值增长7.5%，社会消费品零售总额增长11.5%，地方一般公共预算收入增长8%；城乡人均可支配收入增长高于经济增速，城镇登记失业率控制在4.5%以内，完成省下达的节能减排任务。

（一） 坚决打好三大攻坚战。

一是打好防范化解重大风险攻坚战。突出“五抓五防”，推动重大风险控增减存。加强全口径政府债务管理，推动市县两级融资平台转型。深入开展金融风险排查整治，严厉打击非法集资、金融诈骗等违法犯罪行为，确保陈案化解、新案防控两项指标降低50%。稳妥处置地方法人金融机构经营风险，严控不良贷款增量、加快化解存量，努力实现余额和占比双降。强化重点领域突发事件应急处置。抓好国家安全、意识形态、民族宗教、综治维稳等工作，确保社会大局和谐稳定。

二是打好精准脱贫攻坚战。全面完成建档立卡贫困户和贫困村脱贫出列任务。坚持“志”“智”双扶，增强贫困群众脱贫内生动力和自我发展能力。扎实开展卡外贫困对象、农村饮水安全、最低生活保障和易地扶贫搬迁后续扶持排查。实施农村危房清零、特色产业提升和贫困村提升工程。强化扶贫队伍能力建设，深入开展扶贫领域作风建设，继续加大扶贫财政投入，严格落实扶贫督查考核。有效防控贫困户与非贫困户利益失衡风险。探索完善脱贫攻坚长效机制、脱贫攻坚与乡村振兴衔接机制。

三是打好污染防治攻坚战。抓好中央环保督察反馈问题整改，推进长江大保护十大标志性战役项目化、具体化。落实长江岸线管理员制度。巩固长江干线非法码头整治成果，保持对非法采砂高压严打态势。加快沿江化工企业关改搬转。加强长江沿线排污口巡查核查。实施长江防护林、沿线森林质量提升、腾退岸线植绿复绿等工程，新造林63万亩。加快推进水环境整治工程，全面治理主要水体污水直排，基本消除中心城区黑臭水体。完善省级以上工业园区污水集中处理设施和配套管网，实现园区企业废水全收集、全处理。实施四湖流域水质考核补偿机制。开展重点河流、干渠截污清淤工程，控源截污、清淤疏浚。全面整治饮用水源保护区和自然保护区内排污口。抓好“四个三重大生态工程”，完成厕所革命年度目标任务，推进生活垃圾无害化处理，实现乡镇生活污水处理全覆盖，完成精准灭荒造林任务。确保禁养区内无新增规模化养殖场，禁养区外规模化养殖场一律达标排放。全面落实“一控两减三基本”治理措施，确保农药、化肥使用量分别降低2.3%、3%。深化“三禁两治”，推进全域禁鞭，打好蓝天保卫战，推动空气质量优良天数逐年递增。落实河湖库长制，完善“一河一策”。完成洪湖退垸还湖10万亩。

（二）推动产业高质量发展。

一是提升工业支撑力。抢抓省级战略机遇，推动荆州开发区与江陵工业园区融合发展，谋划建设辐射中部地区和长江沿线城市的煤炭储备交易中心、煤资源深度开发产业中心。完善“一区多园”管理体制，打造千亿产业、千亿园区。滚动实施“千企千亿”技改工程，企业技术改造300家以上，完成技改投资120亿元，力争工业投资增长20%。发展智能制造，推进“千企上云”和重点行业“机器人”应用，智能化改造企业20家，培育省级智能制造试点示范企业2-3家。大力发展高端装备制造、信息技术、新材料等战略性新兴产业。推动军民融合、校地融合，申报省级军民融合示范园，打造石油科技城。实施“百领”企业培育工程，支持重点骨干企业打造行业龙头，新增规模以上工业企业100家。推进多层次资本市场建设，实施“金种子”“银种子”计划，建好上市后备资源库，推动五方光电上市。开展质量提升行动，加快建设质量强市，提升“荆州制造”的含金量。

二是提升农业竞争力。坚持提质增效导向，加快建设农业强市。抓好科技强农，支持荆州高新区打造“中国绿谷”，示范引领全市现代农业发展。加快推进江汉平原农业科技中心、湖北水产产业研究院、监利稻虾生态繁殖基地等平台建设。抓好市场活农，加大市场主体引进和培育力度，新增规上农产品加工企业20家以上。培育新型农业经营主体，加强行业规范化管理，力争农民合作社突破10000家、家庭农场达到9000家以上。支持天露湖创建国家生态农业公园，打造一批集现代农业、休闲度假、田园社区于一体的田园综合体。抓好绿色惠农，推广节肥、节水、节能绿色农业生产技术，探索生态水产养殖模式，扩大“双水双绿”种养规模，新增稻田综合种养面积30万亩以上。抓好质量兴农，叫响“荆州味道”，新申报“三品一标”标志产品20个以上。强化投入品使用监管和农产品质量安全执法，完善质量安全追溯体系，严防、严控、严管农产品质量安全风险，让人民群众吃得放心、吃得安心。

三是提升服务业影响力。抢抓消费升级机遇，优化服务业供给，培育更多新的消费增长点。突破性发展文化旅游业，高位推进荆州历史文化名城保护与利用，打造楚文化、三国文化、水文化交相辉映、特色鲜明的文化旅游板块。全力推进纪南文旅区建设，确保华夏历史文化科技园、园博园建成开园，加快建设荆楚文化大观园、环长湖湿地公园等重点项目。做好“旅游+”文章。完善旅游基础设施，加快建设一批旅游公路。力争旅游综合收入达到360亿元、增长16%。以商圈消费引领商业业态升级。新增限上商贸企业100家。培育商贸品牌，办好荆楚美食节，打造以荆州鱼糕为代表的楚菜精品。评选认定10家荆州“老字号”。大力发展“多式联运”，加快荆州港盐卡综合物流园建设，发展现代供应链管理和冷链物流，建设长江经济带物流节点城市。

四是旗帜鲜明支持民营企业发展。民营经济是荆州经济的主体，民营企业是荆州经济发展的中流砥柱。全面落实国家和省、市惠企政策，减税降费，优化营商环境，让民营企业轻装上阵。加大高端人才引进力度，全面推广新型学徒制，鼓励农民工等人员返乡就业创业，缓解企业“用工难”。着力解决民营企业反映突出的“融资难”问题，促进更多资金流向中小微实体企业。开展金融机构支持民营企业发展评价。创建新型政银担合作模式，为中小微企业提供融资担保15亿元以上。加强区域股权市场融资，为中小微企业提供直接融资20亿元以上。发挥财政资金撬动和引导作用，优化城区实体经济发展调度资金和重大产业基金投向，继续安排续贷调度资金。构建“亲”“清”新型政商关系，切实为企业排忧解难。保护和弘扬爱国敬业、遵纪守法、创业创新、服务社会的企业家精神，引导企业家为地方发展作出更大贡献。

（三）全方位培植后劲支撑。

一是向项目建设要后劲。毫不动摇把项目建设作为经济工作的根本抓手。聚焦长江经济带发展、新旧动能转换、基础设施等领域，抓紧谋划一批重大项目，提升项目库规模和质量。建成荆州开发区绿色建筑产业园、凯乐量子通讯信息安全总部基地、万华零醛智能家居产业园、荆州煤炭储配基地一期、俊力体育用品、一泰科技三期等项目。加快推进恒隆汽车零部件产业园、华中表面处理循环经济产业园、华中体育产业园、华中玻铝产业园、江汉精细化工中德合资项目、锐美智图等项目建设。实施防汛抗旱水利提升工程、水利补短板工程和四湖流域骨干河渠治理工程，强力推进东分块蓄洪工程建设，加速安全转移工程前期工作，完成水利投资20亿元以上。创新投融资方式，推广政府和社会资本合作模式。强化固定资产投资调度，定期开展项目拉练，深化“晒、比、督”机制，完善排名末位发言、争取项目和资金总量公示等制度，形成争先恐后谋项目、争项目、建项目的浓厚氛围。

二是向深化改革要后劲。把优化营商环境作为后发赶超的超车道，用“硬措施”提升“软实力”。深入推进供给侧结构性改革。加快简政放权，继续深化“互联网+政务服务”改革，推动更多政务服务事项“网上办”“掌上办”，力争让群众和企业办事像网购一样方便。全面推行“一次办好、一网覆盖、一站式服务”，“一窗受理”事项达到80%以上，确保政务服务事项网办率达到85%以上、减少办事提供材料70%以上、300个以上高频事项实现“不见面审批”。实现工业建设项目50个工作日内取得施工许可证。深化“证照分离”改革，推广企业登记全程电子化，将企业开办时间压缩到3个工作日。进一步调整权责清单，减少证明事项。全面推进“双随机一公开”，抓好行政执法、刑事司法衔接。因企施策加快国有企业混合所有制改革，促进国有资产保值增值。扎实推进农村集体产权制度改革。持续深化医药卫生体制改革，健全药品供应保障制度，把更多救命救急的好药纳入医保。加快推进分级诊疗制度和县域医共体建设，促进优质医疗资源向基层社区和农村延伸。全面完成机构改革任务。完善信用制度体系，褒扬诚信、惩戒失信，让诚信成为重要的社会资本。

三是向全面开放要后劲。坚持把招商引资作为经济工作的“一号工程”，实施高质量招商行动，新引进亿元以上项目400个以上，各县市区、功能区引进1个50亿元以上项目。围绕支柱产业强链补链，紧盯新一代信息技术、生物医药、新材料等新兴产业，谋划引进一批世界和国内500强、央企、行业龙头等大项目。创新招商方式，用好商会、校友、行业组织等招商资源，强化专业招商、平台招商、产业链招商，提升重资产招商实效。加强对先进制造业、创新型企业的用地保障。加大项目开工建设核查力度，提高合同履约率、项目开工率、建成投产率。大力发展口岸经济，延伸口岸功能，加快打造粮食、肉类等特色口岸。积极对接湖北自贸区。推动国际贸易“单一窗口”建设，开拓外贸市场，力争新开口破零企业25家。培育外贸品牌，建好汽车零部件、化工、纺织品三大出口基地。

四是向创新驱动要后劲。坚持把创新作为引领发展的第一动力，以国家创新型城市建设为引领，强化产业、研发、市场、人才、资本、文化、生态等全要素协同。发展高新技术产业，新增高新技术企业30家，高新增加值占GDP比重达到11.5%以上。增强原始创新能力，积极与省内外高校和科研院所开展合作，新建院士工作站5家，加快建设省油田化学产业技术研究院、省数字出版印刷包装产业研究基地，积极争取设立省级农业产业技术研究院，支持公安、洪湖、监利争创省级农业高新技术产业示范区。发挥企业自主创新主体作用，鼓励引导企业增加研发投入，建成省级企业技术中心2家、工程技术中心3家。对接国家、省重大人才工程，深入实施“我选湖北·凤鸣荆州”计划，加大创新创业战略团队引进力度，鼓励和支持大中专毕业生在荆就业创业。建强科技企业孵化器，完善众创空间、星创天地功能。引导鼓励企业开展科技成果转化，探索金融服务科技成果转化新路径，撬动天使投资、风险投资等社会资本向初创期、种子期企业流动。深化“大众创业、万众创新”，让创业之举遍布荆州大地，让创新之火形成燎原之势。

（四）促进区域统筹城乡融合。

一是优化提升中心城区功能。统筹一城三区，优化城市布局。高标准完成城市总体规划修编。加强对城市整体空间形态、天际线、城市色彩、夜景亮化的规划管理。推进古城人口疏散和功能优化。围绕“畅循环、活水系、拓功能”，实施246个、总投资198亿元的城市基础设施建设项目。建成荆州大道、高阳大道、楚都大道、海子湖大道。加快荆江大道、机场大道等建设。开工环城北路、环城西路、荆江沙市段生态修复与优秀历史建筑保护项目，建成护城河人工湿地及生态驳岸、荆襄外河带状湿地公园，实施城市林荫路、节点景观提升工程。开工建设市民之家、市委党校和荆州殡仪馆搬迁等项目。完成棚改清零。建成8个公共停车场、2600个停车位，启动路内停车经营管理，逐步形成全市统一的智能停车系统。加快实施控违拆违，促进城市管理精细化。持续推进“绿满荆楚”行动，创建国家森林城市。深入开展全国文明城市创建，巩固全国卫生城市创建成果。办好省第二届园林博览会。

二是加快大交通体系建设。全面实施“综合交通三年攻坚行动”，完成交通投资130亿元。建成蒙华铁路、洪监高速、沙公高速北段、潜石高速江石段、荆州长江公铁大桥、石首长江大桥、洪湖嘉鱼长江大桥。建成荆州民用机场主体工程，启动石首市通用机场及航空产业园建设项目。推进李埠长江公铁大桥、观音寺长江大桥、二广高速荆州城区段改线、洪松高速江松段早日开工。谋划荆州南北向高铁通道，争取沿江货运铁路、武贵高铁布局荆州，支持江汉平原铁路建设。实施内荆河航道整治。

三是发展壮大县域经济。鼓励和引导县市区错位竞争、特色发展、争先进位。加强县域实体经济信贷支持。对发展较好、用地集约的县市区实行新增建设用地指标倾斜。支持荆州区建设绿色发展先行区。支持沙市区建设现代服务业集聚区。支持江陵县建设区域性综合交通物流枢纽、中部地区煤炭清洁高效利用示范区。支持松滋市建设湘鄂边重要门户节点城市。支持公安县融入主城区、争创乡村振兴和高质量发展示范县。支持石首市建设链接长江经济带和洞庭湖生态经济区的区域中心城市。支持监利县建设长江中游新兴中等城市。支持洪湖市建设洪湖岸边文旅新城、新滩新城，争创全国水生态文明示范城市。

四是深入实施乡村振兴战略。把乡村振兴作为新时代“三农”工作的总抓手，加快建设江汉平原实施乡村振兴战略示范区。开展以“五整治二提升工程”为重点的农村人居环境整治三年行动，集中整治畜禽粪便、固体废物、河渠沟塘等，新增省级特色小镇1个、生态宜居美丽乡村示范村50个。大力实施“三乡”工程，引导发展要素向乡村流动，增强乡村振兴内生动力。扎实推进“四好农村路”示范县、示范乡镇创建工作，完成“455”安防工程3000公里，改造危桥30座，改扩建农村公路1000公里，建设美丽农村路1000公里。

（五）更好满足人民群众美好生活需要。

一是提升社会保障水平。落实社会保障、医疗卫生、教育等民生政策。全面提高社会救助、孤儿养育、高龄津贴标准。完善经济困难高龄老人、失能老人补贴制度，解决好困难群体和特殊群体基本生存和关爱问题。积极推进医养结合。完善支持大众创业、促进就业政策，新增城镇就业6.6万人以上。进一步扩大住房公积金覆盖面和受益面。加强妇女儿童权益保障。大力发展红十字、残疾人、慈善等事业。通过提升基本公共服务保障能力和水平，进一步增强群众获得感、幸福感。

二是加快发展社会事业。深化校地合作、校地共建，加快推进长江大学东校区南扩。大力发展面向市场、服务发展、促进就业的现代职业教育，为荆州培养更多具有专业技能与工匠精神的人才。科学规划中心城区义务教育布局，推动城乡义务教育均衡发展，推行学区化管理和集团办学。扩大公办幼儿园资源，引导和支持民办幼儿园提供普惠性服务。培育荆州品牌赛事。加强文艺精品创作。强化公共文化服务设施建设，完善基层综合文化中心，完成农村智能广播网升级改造。实施文化惠民工程，推动“悦读书吧”建设，启动“荆州书海”项目，提升人民群众文化素养。落实《“健康荆州2030”行动纲要》，开展健康校园行动。巩固血吸虫病传播阻断达标成果。落实“全面两孩”政策，推进人口持续、均衡发展。积极推进全国食品安全示范城市创建。

三是加强和创新社会治理。坚持共建共治共享，提高社会治理法治化、精细化、智能化、专业化水平。建立健全部门联合执法链条，提升基层治理能力，处理好群众的操心事、烦心事、揪心事，打通服务群众“最后一公里”。建设智慧城市，加快大数据中心建设。鼓励和引导志愿者拓展服务范围、提升服务水平。深入开展“七五”普法，加强公共法律服务平台建设，支持配合检察公益诉讼工作，加快建设法治荆州。深入开展平安荆州创建，加强基层社会治安综合治理中心建设，进一步提升“一感两度两率”。保持扫黑除恶高压态势，切实提升群众满意度。加快推进安全生产领域信息化建设和先进技术推广应用，确保安全生产各项措施落到实处。完善预防和化解社会矛盾机制，及时就地化解矛盾，推进信访法治化。强化国防教育和后备力量建设，巩固军政军民团结良好局面。继续发挥工会、共青团、妇联等群团组织桥梁纽带作用。扎实做好人民防空、防震减灾、民族宗教、外事侨务、拥军优属等工作。

三、加强政府自身建设

各位代表！新的时代，需要新的作为。我们一定不负时代使命、不负人民期望，忠诚干净、实干担当，努力向全市人民交出一份合格答卷。

提高政治站位。树牢“四个意识”，坚定“四个自信”，坚决做到“两个维护”，认真贯彻落实党中央、国务院和省委、省政府的决策部署，坚持以人民为中心的发展思想，紧紧围绕市委确定的目标、思路和重点，研究谋划、贯彻落实，以敢想敢干、善作善成的执行力赢得荆州复兴的加速度。

坚持依法行政。弘扬宪法精神，增强宪法意识，全面落实法治政府建设纲要，把政府活动纳入法治轨道。自觉接受党内监督、人大监督、政协监督、司法监督、舆论监督和社会监督，加强审计监督。坚持重大事项向人大报告、向政协通报，认真办好人大代表建议和政协提案。完善决策机制，推进行政决策科学化、民主化、法治化。

勇于担当作为。强化争先进位意识，推进政府工作目标管理，压实领导责任、牵头责任、推进责任。践行“一线工作法”。紧扣“事前、事中、事后”三环节，打造强有力的“落实链”。跟踪督办、限时办结，以“大督办”倒逼“大落实”。完善问责问效、考评结账等机制，以实效论高低、以实绩论英雄。健全完善激励机制和容错纠错机制，向不作为者亮剑，为实干创新者护航。

厉行廉洁从政。深入贯彻落实全面从严治党和党风廉政建设各项要求，加强源头治理，用制度管权管事管人。加大审计监管力度，严管公共资金，严管公共资源交易，严管国有资本资产。严格控制“三公”经费和一般性支出，把有限的资源和财力用在推动发展、改善民生上。加强政风行风建设，整治形式主义、官僚主义突出问题。政府工作人员要严守政治纪律和政治规矩，严守廉洁从政各项规定，守住底线，不越红线，不碰高压线，努力打造忠诚、干净、担当的公务员队伍。

各位代表！新时代，是奋斗的时代，是奔跑的时代，是追梦圆梦的时代。追梦需要激情和理想，圆梦需要奋斗和奉献。让我们更加紧密地团结在以习近平同志为核心的党中央周围，在省委、省政府和市委的坚强领导下，真抓实干、少说多干、埋头苦干，为加快荆州复兴、决胜全面建成小康社会而不懈奋斗！
